# Characteristic interactivity of landiolol, an ultra-short-acting highly selective β_1_-blocker, with biomimetic membranes: Comparisons with β_1_-selective esmolol and non-selective propranolol and alprenolol

**DOI:** 10.3389/fphar.2013.00150

**Published:** 2013-12-02

**Authors:** Hironori Tsuchiya, Maki Mizogami

**Affiliations:** ^1^Department of Dental Basic Education, Asahi University School of DentistryMizuho, Gifu, Japan; ^2^Department of Anesthesiology and Reanimatology, University of Fukui Faculty of Medical SciencesEiheiji-cho, Fukui, Japan

**Keywords:** landiolol, selective β_1_-blocker, membrane interactivity, biomimetic membrane, antioxidant activity

## Abstract

Although β_1_-blockers have been perioperatively used to reduce the cardiac disorders associated with general anesthesia, little is known about the mechanistic characteristics of ultra-short-acting highly selective β_1_-blocker landiolol. We studied its membrane-interacting property in comparison with other selective and non-selective β_1_-blockers. Biomimetic membranes prepared with phospholipids and cholesterol of varying compositions were treated with β_1_-selective landiolol and esmolol and non-selective propranolol and alprenolol at 0.5–200 μM. The membrane interactivity and the antioxidant activity were determined by measuring fluorescence polarization and by peroxidizing membrane lipids with peroxynitrite, respectively. Non-selective β_1_-blockers, but not selective ones, intensively acted on 1,2-dipalmitoylphosphatidylcholine (DPPC) liposomal membranes and cardiomyocyte-mimetic membranes to increase the membrane fluidity. Landiolol and its inactive metabolite distinctively decreased the fluidity of DPPC liposomal membranes, suggesting that a membrane-rigidifying effect is attributed to the morpholine moiety in landiolol structure but unlikely to clinically contribute to the β_1_-blocking effect of landiolol. Propranolol and alprenolol interacted with lipid raft model membranes, whereas neither landiolol nor esmolol. All drugs fluidized mitochondria-mimetic membranes and inhibited the membrane lipid peroxidation with the potency correlating to their membrane interactivity. Landiolol is characterized as a drug devoid of the interactivity with membrane lipid rafts relating to β_2_-adrenergic receptor blockade. The differentiation between β_1_-blocking selectivity and non-selectivity is compatible with that between membrane non-interactivity and interactivity. The mitochondrial membrane fluidization by landiolol independent of blocking β_1_-adrenergic receptors is responsible for the antioxidant cardioprotection common to non-selective and selective β_1_-blockers.

## INTRODUCTION

Noxious stimuli by anesthesia induction, operative incision, laryngoscopy, tracheal intubation, and/or extubation excite the sympathetic nervous system, resulting in heart rate increase, arterial blood pressure elevation, and cardiac ischemia occurrence. The perioperative use of β-adrenergic receptor antagonists has been suggested to reduce the risk of such heart events as tachycardia, hypertension, myocardial ischemia and infarction and the surgery-relating cardiac morbidity and mortality during general anesthesia (Devereaux et al., 2005; Wiesbauer et al., 2007; Zangrillo et al., 2009). In addition, β-blockers show the antinociceptive property to decrease intraoperative anesthetic and analgesic requirements (Davidson et al., 2001) and the blocking effects on voltage-gated sodium channels (Wang et al., 2010).

Propranolol was previously used as a pre-, intra-, and postoperative β-blocker (Ivey et al., 1983; Wiesbauer et al., 2007), followed by oxprenolol, labetalol, nadolol, timolol, and alprenolol (Burns et al., 1988; Fleisher et al., 2009). However, these conventional drugs have the possibility to cause long-lasting cardiac failures and respiratory side-effects due to their concomitant β_2_-blocking effects. Although cardioselective β-blockers such as atenolol and metoprolol were alternatively used, their duration and intensity of action were problematic for the perioperative use, leading to the development of short-acting β_1_-selective esmolol with the selectivity of β_1_/β_2_ = 33 and the half-life (*t*_1/2_) = 9.19 min (Sum et al., 1983). The subsequent studies produced ultra-short-acting highly β_1_-selective landiolol with the selectivity of β_1_/β_2_ = 255 and the half-life (*t*_1/2_) = 3.96 min (Iguchi et al., 1992). Landiolol and esmolol show Ki values of 62/1890 nM and 125/2620 nM in human β_1_-/β_2_-adrenergic receptors and 993/12416 nM and 1054/5900 nM in dog β_1_-/β_2_-adrenergic receptors, indicating that the β_1_-selectivity relative to propranolol is 74–380 for landiolol and 39–263 for esmolol (Japan Pharmaceutical Information Center [JAPIC], 2012). These sophisticated β_1_-blockers have been evaluated as an agent suitable for perioperative tachycardia and hypertension without the risk of prolonged cardiac depression but with the benefit to decrease anesthetic requirements (Saito et al., 2005; Tanabe et al., 2009).

The selectivity of antagonists is exclusively attributed to their structure-specific binding to receptors embedded in biomembranes. Besides receptor proteins, however, β-blockers also act on membrane lipids to modify the physicochemical property of biomembranes such as fluidity (Varga et al., 1999; Lombardi et al., 2009). Because lipid bilayers provide transmembrane receptors with the surrounding environments optimal for their activity, changes in membrane fluidity influence the β-adrenergic receptor signaling (Ma et al., 1997). The property to change membrane fluidity has been suggested for several drugs acting on β-adrenergic receptors (Butler et al., 2006; Lombardi et al., 2009). Conventional β_1_-blockers possess the ability to interact with lipid bilayer membranes (Varga et al., 1999; Pereira-Leite et al., 2013). The membrane-interacting characteristics including potency and selectivity were recently reported to be useful for differentiating between non-selective β_1_-blockers (including propranolol, alprenolol, and oxprenolol) and selective β_1_-blockers (including atenolol, metoprolol, and esmolol; Mizogami et al., 2010).

Although both landiolol and esmolol are classified as a short-acting β_1_-selective blocker, they are different in pharmacological features (Iguchi et al., 1992; Saito et al., 2005). However, there have been no investigations on the membrane effects to characterize landiolol despite that its structurally relating or structural moiety-containing compound acts on lipid membranes (Tian et al., 2011). In order to provide a novel pharmacological insight into landiolol, we studied its interactivity with different kinds of biomimetic membranes by comparing with β_1_-selective esmolol and non-selective propranolol and alprenolol.

## MATERIALS AND METHODS

### REAGENTS

**FIGURE 1:**
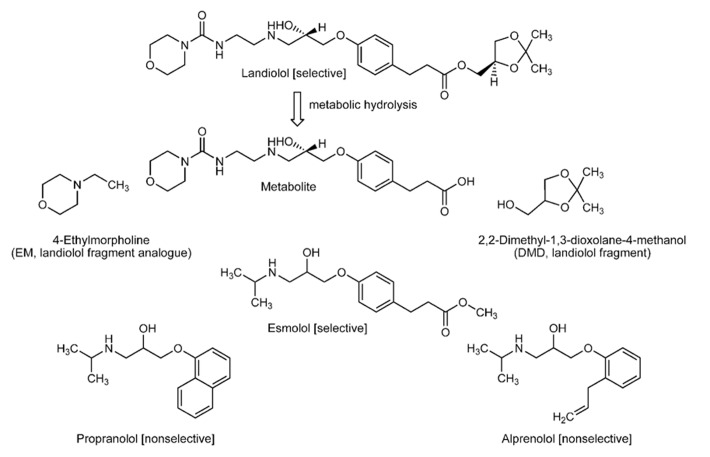
**Structures of selective and non-selective β _**1**_-blockers and landiolol-related compounds**.

Landiolol ((–)-[(*S*)-2,2-dimethyl-1,3-dioxolan-4-yl]methyl 3-{4-[(*S*)-2-hydroxy-3-(2-morpholinocarbonylamino)ethylamino] propoxy}phenylpropionate) and its metabolite (3-{4-[(*S*)-2-hydroxy-3-(2-morpholinocarbonylamino)ethylamino]propoxy}phenylpropionic acid) were supplied by Ono Pharmaceuticals (Osaka, Japan), and esmolol by Maruishi Pharmaceuticals (Osaka, Japan). Propranolol and alprenolol were purchased from Sigma-Aldrich (St. Louis, MO, USA), and 4-ethylmorpholine (EM) and 2,2-dimethyl-1,3-dioxolane-4-methanol (DMD) from Tokyo Chemical Industrials (Tokyo, Japan). Their chemical structures are shown in Figure 1. 1,2-Dipalmitoylphosphatidylcholine (DPPC), 1-palmitoyl-2-oleoylphosphatidylcholine (POPC), 1,2-dioleoylph osphatidylcholine (DOPC), 1-palmitoyl-2-oleoylphosphatidyleth anolamine (POPE), 1-palmitoyl-2-oleoylphosphatidylserine (POPS), bovine heart cardiolipin (CL), porcine brain phosphatidylinositol (PI), porcine brain sphingomyelin (SM), and porcine brain cerebroside (CB) were purchased from Avanti Polar Lipids (Alabaster, AL, USA), and cholesterol and α-tocopherol from Wako Pure Chemicals (Osaka, Japan). 1,6-Diphenyl-1,3,5-hexatriene (DPH) was obtained from Molecular Probes (Eugene, OR, USA), and diphenyl-1-pyrenylphosphine (DPPP) and peroxynitrite from Dojindo (Kumamoto, Japan). Dimethyl sulfoxide (DMSO) of spectroscopic grade (Kishida, Osaka, Japan) was used for preparing reagent solutions.

### MEMBRANE PREPARATION

Biomimetic membranes labeled with DPH were prepared with phospholipids and cholesterol to be unilamellar vesicles suspended in a buffer as reported previously (Tsuchiya and Mizogami, 2008). In brief, an aliquot (250 μl) of the ethanol solution of phospholipids and cholesterol (total lipids of 10 mM) and DPH (50 μM) was injected four times into 199 ml of 10 mM HEPES (4-(2-hydroxyethyl)-1-piperazineethanesulfonic acid) buffer of pH 7.4 containing 125 mM NaCl and 25 mM KCl under stirring above the phase transition temperatures of phospholipids. The membrane lipid compositions were as follows: (1) 100 mol% DPPC for DPPC liposomal membranes which have been most frequently used in membrane interaction experiments (Mizogami et al., 2010; Pereira-Leite et al., 2013), (2) 25 mol% POPC, 20 mol% POPE, 5 mol% POPS, 5 mol% PI, 5 mol% SM, and 40 mol% cholesterol for cardiomyocyte-mimetic membranes (Wheeldon et al., 1965), (3) 16.7 mol% DOPC, 16.7 mol% POPE, 16.7 mol% SM, 16.7 mol% CB, and 33.3 mol% cholesterol for lipid raft model membranes (Schroeder et al., 1994) and (4) 25 mol% POPC, 16 mol% POPE, 3 mol% POPS, 10 mol% CL, 3 mol% PI, 3 mol% SM, and 40 mol% cholesterol for mitochondria-mimetic membranes (Tsuchiya et al., 2010a).

### MEMBRANE INTERACTIVITY

The membrane interactivity was determined by analyzing the drug-induced changes in membrane fluidity as reported previously (Tsuchiya et al., 2011). In brief, landiolol, its metabolite, its structurally relating compounds (EM and DMD), esmolol, propranolol, and alprenolol were dissolved in DMSO. The resulting solutions were applied to the membrane preparations so that a final concentration of drugs was 0.5–200 μM. These drug concentrations were chosen because the tested β_1_-blockers were reported to show blood concentrations of a micromolar level in their pharmacokinetic studies (de Bruijn et al., 1987; Murakami et al., 2005). The concentration of DMSO was adjusted to be 0.25% (v/v) of the total volume so as not to affect the fluidity of intact membranes. Beta_1_-selective esmolol and non-selective propranolol and alprenolol were used for the comparisons because they have the structurally same substituent (2-hydroxy-3-(isopropylamino)propoxyl group) attached to aromatic rings (see Figure 1). Control experiments were conducted with the application of an equivalent volume of DMSO vehicle. After the reaction at 37^°^C for 30 min, DPH fluorescence polarization was measured by an RF-540 spectrofluorometer (Shimadzu, Kyoto, Japan) equipped with a polarizer at excitation 360 nm and at emission 430 nm as reported previously (Mizogami et al., 2010). Polarization values were calculated by the formula (*I*_VV_ - *GI*_VH_)/(*I*_VV_ + *GI*_VH_) according to the method of Ushijima et al. (2005), in which *I* is the fluorescence intensity and the subscripts V and H refer to the vertical and horizontal orientation of excitation and emission polarizer, respectively. The grating correction factor (*G* = *I*_HV_/*I*_HH_) is the ratio of the detection system sensitivity for vertically and horizontally polarized light, which was used to correct the polarizing effects of a monochromator. Decreasing and increasing polarization changes from controls mean an increase (membrane fluidization) and a decrease of membrane fluidity (membrane rigidification), respectively.

### ANTIOXIDANT ACTIVITY

The antioxidant activity to inhibit membrane lipid peroxidation was determined by the liposomal system as reported previously (Tsuchiya et al., 2010b). In brief, DPPP-incorporated membranes with the molar ratio of DPPP to total membrane lipids of being 1:100 were prepared to be liposomes suspended in Dulbecco’s phosphate-buffered saline of pH 7.4 (Dainippon Pharmaceuticals, Osaka, Japan). Their membrane lipid compositions were (1) 100 mol% DOPC for unsaturated phospholipid membranes and (2) 25 mol% POPC, 16 mol% POPE, 3 mol% POPS, 10 mol% CL, 3 mol% PI, 3 mol% SM, and 40 mol% cholesterol for mitochondria-mimetic membranes (Tsuchiya et al., 2010a). Liposome suspensions of 3.97 ml were pre-incubated at 37^°^C for 30 min with each 10 μl of selective and non-selective β_1_-blocker solutions in DMSO (a final concentration of 100 μM for each drug) or the α-tocopherol solution in DMSO (2.5 μM) as a reference antioxidant. A corresponding volume (0.25%, v/v) of DMSO vehicle was added to controls. Lipid peroxidation was induced by adding 20 μl of the peroxynitrite solution in 0.1 M NaOH (a final concentration of 20 μM) and then incubating at 37^°^C for 10 min. Since membrane-incorporated DPPP quantitatively reacted with a lipid hydroperoxide to produce a fluorescent phosphine oxide, the liposome suspensions were fluorometrically analyzed at excitation 355 nm and at emission 382 nm. When the peroxynitrite-induced increase in fluorescence intensity reached a plateau, membrane lipid peroxidation was defined as completed (100%). The lipid peroxidation-inhibiting percentages were determined by comparing the fluorescence intensity with controls. Because DMSO has the antioxidant property to potentially inhibit lipid peroxidation (Sanmartín-Suárez et al., 2011), it may cooperatively increase the lipid peroxidation-inhibitory effects of the tested drugs. In the present study, the fluorescence intensity of liposomes treated with DMSO alone was subtracted from that of liposomes treated with drugs plus DMSO so that the determined activity was not influenced by DMSO.

### STATISTICAL ANALYSIS

All results are expressed as means ± SEM (*n* = 8 for membrane interactivity experiments and *n* = 5 for antioxidant activity experiments). Data were analyzed by a one-way analysis of variance (ANOVA) followed by a *post hoc* Fisher’s protected least significant difference (PLSD) test using StatView version 5.0 (SAS Institute, Cary, NC, USA). A *p* value of being < 0.05 was taken as significant.

## RESULTS

### INTERACTION WITH BIOMIMETIC MEMBRANES

**FIGURE 2:**
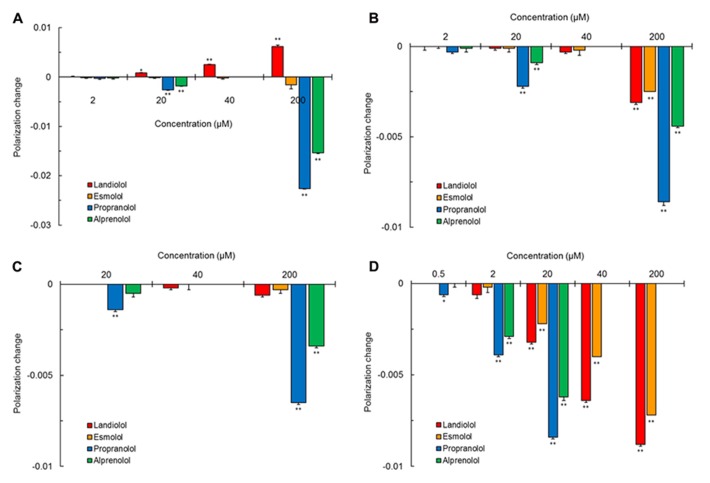
**Interaction of selective and non-selective β_1_-blockers with different kinds of biomimetic membranes.** All drugs were reacted at the indicated concentrations with 100 mol% DPPC liposomal membranes **(A)**, cardiomyocyte-mimetic membranes **(B)**, lipid raft model membranes **(C)**, and mitochondria-mimetic membranes **(D)**, followed by measuring DPH fluorescence polarization. Values represent means ± SEM (*n* = 8). *p < 0.05 and **p < 0.01 vs. control.

Propranolol and alprenolol interacted with different membrane preparations to increase the fluidity of all of them as shown by polarization decreases in Figure 2. These non-selective β_1_-blockers fluidized DPPC liposomal membranes (Figure 2A), cardiomyocyte-mimetic membranes (Figure 2B) and lipid raft model membranes (Figure 2C) at 20–200 μM and mitochondria-mimetic membranes (Figure 2D) at lower concentrations of 0.5–20 μM. In contrast, selective β_1_-blockers so differently acted on DPPC liposomal membranes that landiolol decreased the membrane fluidity at 20–200 μM as shown by polarization increases, but not esmolol (Figure 2A). Landiolol and esmolol induced much less fluidization in cardiomyocyte-mimetic membranes (Figure 2B) and no fluidization in lipid raft model membranes (Figure 2C) even at 200 μM. However, both selective β_1_-blockers interacted with mitochondria-mimetic membranes to fluidize them at 20–200 μM as well as non-selective propranolol and alprenolol (Figure 2D).

### MEMBRANE EFFECTS OF LANDIOLOL AND RELATED COMPOUNDS

**FIGURE 3:**
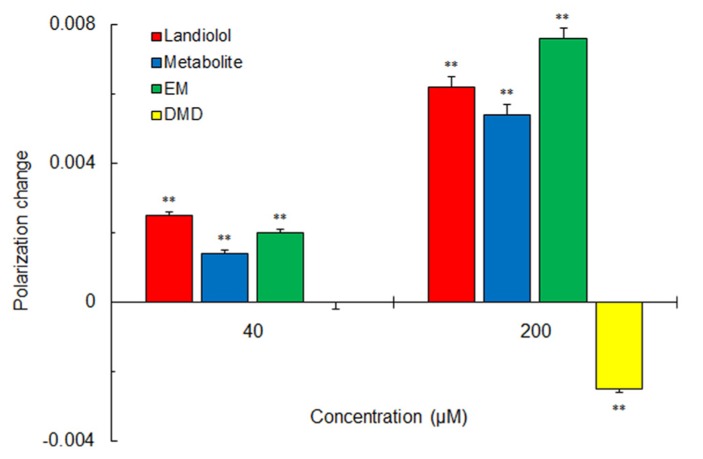
**Effects of landiolol, its hydrolysis metabolite and structural fragments (40 and 200 μM for each) on 100 mol% DPPC liposomal membranes.** Values represent means ± SEM (*n* = 8). **p < 0.01 vs. control.

Not only landiolol but its metabolite and a hydrolysis fragment analog EM rigidified DPPC liposomal membranes (Figure 3). However, another hydrolysis fragment DMD was not effective in rigidifying the membranes or reversely fluidized the membranes at a relatively high concentration.

### ANTIOXIDANT EFFECTS ON BIOMIMETIC MEMBRANES

**FIGURE 4:**
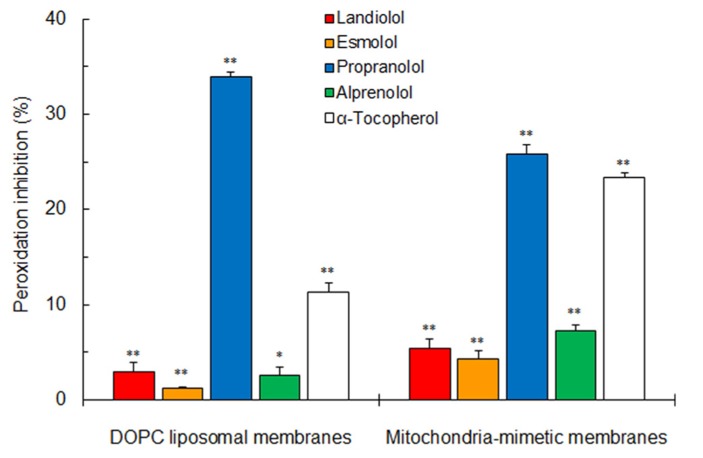
**Inhibitory effects of selective and non-selective β_**1**_-blockers (100 μM for each) and antioxidant α-tocopherol (2.5 μM) on peroxynitrite-induced lipid peroxidation of 100 mol**%** DOPC liposomal membranes and mitochondria-mimetic membranes.** Values represent means ± SEM (*n* = 5). *p < 0.05 and **p < 0.01 vs. control.

Both selective and non-selective β_1_-blockers inhibited the peroxynitrite-induced peroxidation of DOPC liposomal membranes and mitochondria-mimetic membranes as well as antioxidant α-tocopherol (Figure 4). Propranolol was greatest in antioxidant activity on biomimetic membranes, followed by alprenolol, landiolol, and esmolol in the decreasing order of potency.

## DISCUSSION

Pereira-Leite et al. (2013) used different fluorescence probes DPH and 1-(4-trimethylammoniumphenyl)-6-phenyl-1,3,5-hexatriene (TMA-DPH) for comparing the membrane interactivity of non-selective and selective β_1_-blockers. All the drug-induced polarization changes were much greater in DPH than in TMA-DPH. DPH is localized in the hydrocarbon core of lipid bilayers to show the fluidity change in deeper membrane regions, whereas TMA-DPH is anchored at the polar head groups of phospholipids to show the fluidity change in surface membrane regions (Tsuchiya, 2001). Both non-selective and selective β_1_-blockers are considered to preferentially interact with the hydrophobic acyl chain regions of phospholipid membranes. Therefore, we used DPH for determining the membrane interactivity of landiolol and reference drugs. Our main findings are as follows: (1) propranolol and alprenolol interact with DPPC liposomal, cardiomyocyte-mimetic, lipid raft model, and mitochondria-mimetic membranes to fluidize all of them at sub-μM or μM concentrations, although landiolol and esmolol are not so interactive with cardiomyocyte-mimetic and lipid raft model membranes, (2) only landiolol rigidifies DPPC liposomal membranes in contrast to membrane-fluidizing propranolol and alprenolol or membrane-inactive esmolol, and (3) both non-selective and selective β_1_-blockers interact with mitochondria-mimetic membranes to increase their fluidity together with inhibiting the peroxynitrite-induced lipid peroxidation of biomimetic membranes.

Beta_1_-blockers are structurally composed of an aromatic ring and a 2-hydroxy-3-(isopropylamino)propoxyl group or its structural analog. Alprenolol is the phenyl derivative with a 2-hydroxy-3-(isopropylamino)propoxyl group and a 2-propenyl group at the *ortho*-position and propranolol has a bulky α-naphthalene nucleus with a 2-hydroxy-3-(isopropylamino)propoxyl group. Such molecular structures of non-selective β_1_-blockers occupy more space in membrane lipid bilayers with the resultant perturbation of the alignment of phospholipid acyl chains, thereby inducing fluidity changes in biomimetic membranes. On the other hand, landiolol and esmolol have two side chains in the *para*-positions. Therefore, they show an almost linear configuration in membrane lipid bilayers which allows drug molecules to align approximately parallel to phospholipid acyl chains. Due to such an alignment, these selective β_1_-blockers could not induce significant changes in membrane fluidity even if penetrating into cardiomyocyte-mimetic and lipid raft model membranes (Mizogami et al., 2010).

Landiolol characteristically acted on DPPC liposomal membranes to rigidify them. Its metabolite lacking a DMD substructure and its hydrolysis fragment analog EM also rigidified DPPC liposomal membranes, but not landiolol hydrolysis fragment DMD, suggesting that the morpholine moiety provides landiolol with a rigidifying effect on DPPC membranes. Landiolol is metabolically hydrolyzed by esterase in plasma and liver and the resulting metabolite is pharmacologically inactive. Biological membranes are composed of different phospholipids and cholesterol, not of DPPC alone. Although the action on DPPC membranes is of much interest as a unique physicochemical property of landiolol, it is unlikely to clinically contribute to blocking β_1_-adrenergic receptors.

A recent concept on biomembranes has indicated that they are not a simple bilayer structure of uniformly distributed lipids but contain the microdomain lipid rafts biophysically different from bulk membranes (Simons and Toomre, 2000). Highly ordered membrane microdomains encompass β-adrenergic receptors and provide them with the platform to regulate their functions (Lanoul et al., 2005). Lipid rafts form caveolae by polymerizing with caveolins which bind to cholesterol. The localization in caveolae/lipid rafts is prerequisite to β_2_-adrenergic receptors for physiologic signaling, but not to β_1_-adrenergic receptors (Xiang et al., 2002). Propranolol and alprenolol act on lipid raft model membranes and fluidize them. Membrane fluidization is associated with the decreased function of β_2_-adrenergic receptors (Lombardi et al., 2009). Non-selective β_1_-blockers would reduce the β_2_-adrenergic receptor activity by interacting with membrane lipid rafts together with antagonizing β_1_-adrenergic receptors by binding to β_1_-receptor proteins, thereby producing the non-selective blockade. Their effects on cardiomyocyte membranes may also contribute to blocking β_2_-adrenergic receptors. Because neither landiolol nor esmolol interact with lipid raft model membranes, these selective β_1_-blockers could not influence the β_2_-adrenergic receptor activity through membrane fluidization, enhancing the selectivity to β_1_-adrenergic receptor blockade. The differentiation between selectivity and non-selectivity to β_1_-adrenergic receptors is compatible with that between non-interactivity and interactivity with biomimetic membranes, which is consistent with the previous comparisons between selective (atenolol, metoprolol, esmolol) and non-selective β_1_-blockers (alprenolol, oxprenolol, propranolol; Mizogami et al., 2010). A correlation between membrane interaction and low β_1_-specificity is likely to apply to most non-selective drugs. Unlike β_1_-non-selective propranolol, β_1_-selective landiolol and esmolol show no interactions with lipid raft model membranes or much less interactivity with cardiomyocyte-mimetic membranes. The β_1_-selectivity associated with the membrane non-interactivity is consistent with the relative β_1_-selectivity of landiolol (74–380), esmolol (33–263), and propranolol (1) reported previously (Sum et al., 1983; Iguchi et al., 1992; Japan Pharmaceutical Information Center [JAPIC], 2012).

Both non-selective and selective β_1_-blockers not only interact with mitochondria-mimetic membranes to increase their fluidity but also inhibit lipid peroxidation of DOPC liposomal membranes and mitochondria-mimetic membranes. In this study, mitochondria-mimetic membranes were prepared to contain 10 mol% CL. CL is preferentially located in cardiac mitochondrial membranes to play an important role in heart functions and it comprises 8–20% of total mitochondrial phospholipids in cardiomyocytes (Houtkooper and Vaz, 2008). CL has two negatively charged head groups, whereas the side chains of all the tested β_1_-blockers have a positively chargeable imino structure. Cationic non-selective and selective β_1_-blockers appear to electrostatically interact with anionic CL in membrane lipid bilayers (Tsuchiya et al., 2010a). Such an interaction accounts for their greater effects on mitochondria-mimetic membranes compared with cardiomyocyte-mimetic membranes not containing CL.

Reactive oxygen species are produced during various cardiac disorders (Paradies et al., 2004). Nitric oxide and superoxide anion rapidly react to generate peroxynitrite which is pathologically responsible for cardiac ischemia-reperfusion injury, surgery-relating complication, and cardiovascular damage through the lipid peroxidation of biomembranes (Lalu et al., 2002). When lipid peroxidation is induced by peroxynitrite, the rank order of antioxidant activity (propranolol > alprenolol > landiolol > esmolol) agrees with that of mitochondria-mimetic membrane interactivity. The modification of membrane fluidity is mechanistically associated with the inhibition of membrane lipid peroxidation (Saija et al., 2001; Lúcio et al., 2007). Radical and antioxidant molecules are likely to interact more efficiently in fluidized membrane lipid environments (Tsuchiya et al., 2010b; Pereira-Leite et al., 2013). Since reactive oxygen species peroxidize cell membranes to produce myocardial ischemia/reperfusion damages, the reduction of membrane lipid peroxidation leads to the protection of hearts (Kimura-Kurosawa et al., 2007). The antioxidant activity not directly relating to β-adrenergic receptor blockade has been indicated to underlie the cardioprotective effects of β-blockers (Kramer et al., 2006). Landiolol, esmolol, propranolol, and alprenolol would exert the cardioprotection by their common membrane-fluidizing property distinct from the β_1_-adrenergic receptor-blocking one.

The clinical implications of the membrane interaction of β_1_-blockers may be argued about their relevant concentrations to modify membrane fluidity. The concentrations of landiolol, esmolol, and propranolol to inhibit membrane lipid peroxidation almost correspond to those to protect from the ischemia-reperfusion injury (Kurosawa et al., 2003). Hydrophobic β_1_-blockers are concentrated in membrane lipid bilayers and intracellularly accumulated over 1000 times higher than their incubation medium concentrations (Butler et al., 2006; Kramer et al., 2006).

## CONCLUSION

To our knowledge, this is the first study to determine the membrane interactivity of landiolol depending on the lipid composition of biomimetic membranes. Landiolol is characterized by the non-interactivity with membrane lipid rafts which enhances its selectivity to β_1_-adrenergic receptor blockade. On the other hand, landiolol is able to interact with CL-containing mitochondrial membranes to increase the membrane fluidity as well as propranolol, alprenolol, and esmolol. Its lipid peroxidation-inhibitory effect associated with membrane fluidization would produce the clinical benefit of cardioprotection common to non-selective and selective β_1_-blockers by the mechanism independent of blocking β_1_-adrenergic receptors.

## Conflict of Interest Statement

The authors declare that the research was conducted in the absence of any commercial or financial relationships that could be construed as a potential conflict of interest.

## AUTHOR CONTRIBUTIONS

Hironori Tsuchiya: Designed the study, conducted the study, and wrote the manuscript. Maki Mizogami: Performed the experiments, analyzed the data, and wrote the manuscript.

## ABBREVIATIONS

CB: cerebroside
CL: cardiolipin
DMD: 2,2-dimethyl-1,3-dioxolane-4-methanol
DMSO: dimethyl sulfoxide
DOPC: 1,2-dioleoylphosphatidylcholine
DPH: 1,6-diphenyl-1,3,5-hexatriene
DPPC: 1,2-dipalmitoylphosphatidylcholine
DPPP: diphenyl-1-pyrenylphosphine
EM: 4-ethylmorpholine
PI: phosphatidylinositol
POPC: 1-palmitoyl-2-oleoylphosphatidylcholine
POPE: 1-palmitoyl-2-oleoylphosphatidylethanolamine
POPS: 1-palmitoyl-2-oleoylphosphatidylserine
SM: sphingomyelin
TMA-DPH: 1-(4-trimethylammoniumphenyl)-6-phenyl-1,3,5-hexatriene
